# After Thirty Years, We Still Cannot Understand Why Methylene Blue is not a Reference to Treat Vasoplegic Syndrome in Cardiac Surgery

**DOI:** 10.21470/1678-9741-2021-0955

**Published:** 2021

**Authors:** Paulo Roberto B. Evora, Ricardo O. S. Soares, Solange Bassetto, Maria Auxiliadora Martins, Fábio Luis da Silva Silva, Anibal Basile Filho

**Affiliations:** 1 Department of Surgery & Anatomy, Faculdade de Medicina de Ribeirão Preto, Universidade de São Paulo, Ribeirão Preto, São Paulo, Brazil.; 2 Faculdade de Medicina de Marília, Marília, São Paulo, Brazil.

**Keywords:** Vasoplegia, Cardiac Surgical Procedures, Methylene Blue, Nitric Oxide, Neutrophils, Hypotension

## Abstract

Vasoplegic syndrome (VS) comprises a constellation of concurrent signs and symptoms: hypotension, high cardiac index, low systemic vascular resistance, low filling pressures, the tendency to occur diffuse bleeding, and sustained hypotension. All of these parameters may persist even despite the use of high doses of vasoconstrictor amines. VS arises from vasoplegic endothelial dysfunction with excessive release of nitric oxide by polymorphonuclear leukocytes mediated by the nitric oxide synthase’s inducible form and is associated with systemic inflammatory reaction and high morbimortality. The achievements regarding the treatment of VS with methylene blue (MB) are a valuable Brazilian contribution to cardiac surgery. The present text review was designed to deliver the accumulated knowledge in the past ten years of employing MB to treat VS after cardiac surgery. Considering that we have already published two papers describing acquired experiences and concepts after 15 and 20 years, now, as we achieve the 30-year mark, we compose a trilogy.

**Table:** 

Abbreviations, acronyms & symbols				
**5-HT**	**= 5-hydroxytryptamine**		**LPS**	**= Lipopolysaccharide**
**ADP**	**= Adenosine diphosphate**		**MAP**	**= Mean arterial pressure**
**ATP**	**= Adenosine triphosphate**		**MB**	**= Methylene blue**
**cAMP**	**= 3',5'-cyclic adenosine monophosphate**		**NO**	**= Nitric oxide**
**cGMP**	**= Cyclic guanosine monophosphate**		**NOS**	**= Nitric oxide synthase**
**EDRFs**	**= Endothelium-derived relaxing factors**		**PGH_2_**	**= Prostaglandin H2**
**eNOS**	**= Endothelial nitric oxide synthase**		**PGI_2_**	**= Prostacyclin**
**Gp**	**= Gp proteins**		**PIP_2_**	**= Phosphatidylinositol 4,5-biphosphate**
**GS**	**=Gs proteins**		**PLC**	**= Phospholipase C**
**GTP**	**= Guanosine triphosphate**		**R**	**= Receptor**
**iNOS**	**= Inducible nitric oxide synthase**		**sGC**	**= Soluble guanylyl cyclase**
**IP_3_**	**= Inositol triphosphate**		**VS**	**= Vasoplegic syndrome**
**IV**	**= Intravenous**		**TXA_2_**	**= Thromboxane A2**

## INTRODUCTION

**Fig. 1:**
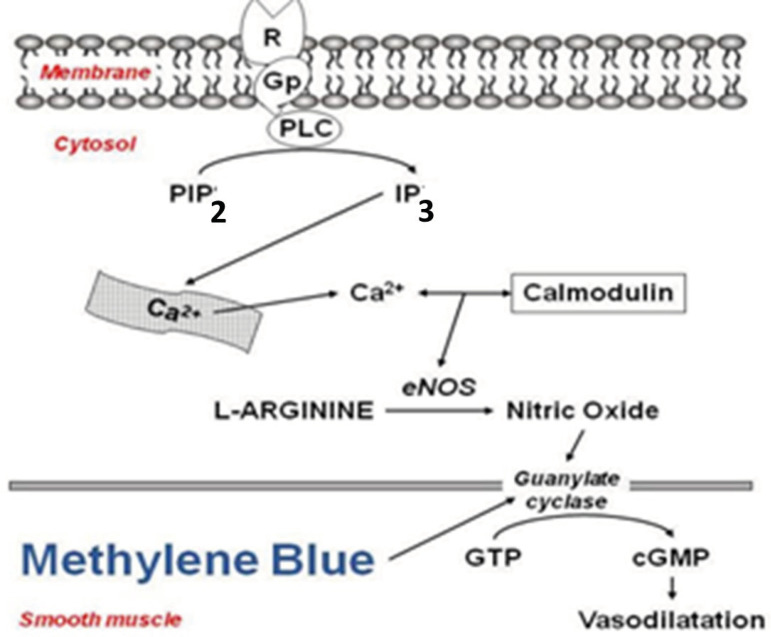
The classical endothelial nitric oxide (NO) release pathway requires 1) signal transduction from a cell receptor-mediated by G proteins (Gp); 2) phospholipase C (PLC) activation and production of inositol triphosphate (IP3) from phosphatidylinositol 4,5-biphosphate (PIP2) and cytosolic Ca2+ release; 3) the constitutive endothelial NO synthase (eNOS) is activated by complex Ca2+/calmodulin and produces NO from its substrate L-arginine; 4) NO stimulates guanylate cyclase in adjacent smooth muscle cells, which causes an increase in cyclic guanosine monophosphate (cGMP) that is the final stimulus that causes vasorelaxation; and 5) methylene blue inhibits the activity of guanylate cyclase, decreasing the cGMP production and less vascular smooth muscle relaxation (adapted from Evora et al., 2009[^1^]). GTP=guanosine triphosphate; R=receptor

**Fig. 2:**
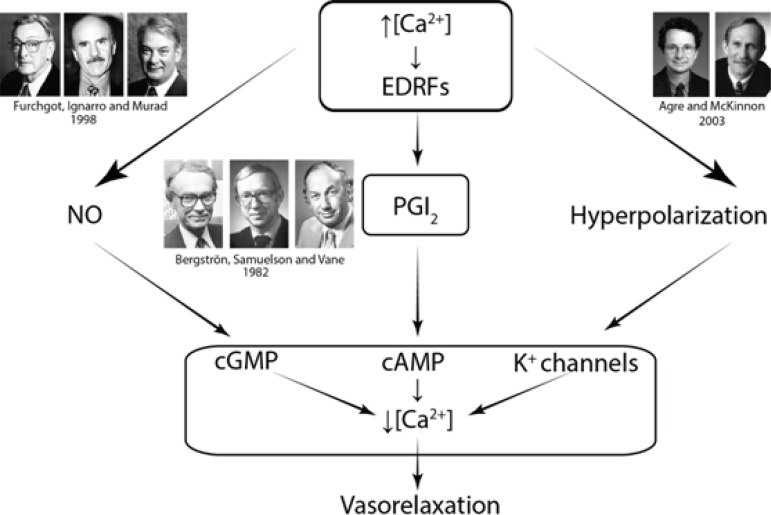
The three mechanisms of vasodilatation. cAMP=3’,5’-cyclic adenosine monophosphate; cGMP=cyclic guanosine monophosphate; EDRFs=endothelium-derived relaxing factors; NO=nitric oxide; PGI2=prostacyclin

Vasoplegic syndrome (VS) comprises a constellation of concurrent signs and symptoms: hypotension, high cardiac index, low systemic vascular resistance, low filling pressures, the tendency to occur diffuse bleeding, and sustained hypotension. All of these parameters may persist even despite the use of high doses of vasoconstrictor amines. VS arises from vasoplegic endothelial dysfunction with excessive release of nitric oxide (NO) by polymorphonuclear leukocytes, mediated by the inducible form of the NO synthase (NOS), and is associated with systemic inflammatory reaction and high morbimortality. The discovery of NO as a mediator of vascular reactivity brought about a paradigm shift, revolutionizing the understanding and searching for new frontiers in cardiocirculatory pathophysiology. According to the words of Salvador Moncada, it created a situation in which everything that was learned about the entity “vasoconstriction” should be reviewed, considering the phenomenon as a “loss of vasodilation” rather than simple constriction of the blood vessels. This review’s primary purpose does not intend to include the “NO saga” in more detail; however, for didactic purposes, we chose to include a schematic representation of the cyclic guanosine monophosphate (cGMP)/NO release pathway (Figure 1). The present review focuses only on the VS associated with cardiac surgery, considering the three vasodilation mechanisms known and awarded with the Nobel Prize (Figure 2).

A summarized historical review shows that the definition of VS and its treatment are valuable Brazilian contributions to cardiac surgery. Gomes^[1]^ described the syndrome, and the treatment with methylene blue (MB) was proposed by Evora et al.^[2]^. The first documentation of this therapeutic approach in patients who underwent heart surgery was presented by Andrade et al.^[3]^, in 1996, at a Congress of the Sociedade Brasileira de Cirurgia Cardiovascular.

**Fig. 3:**
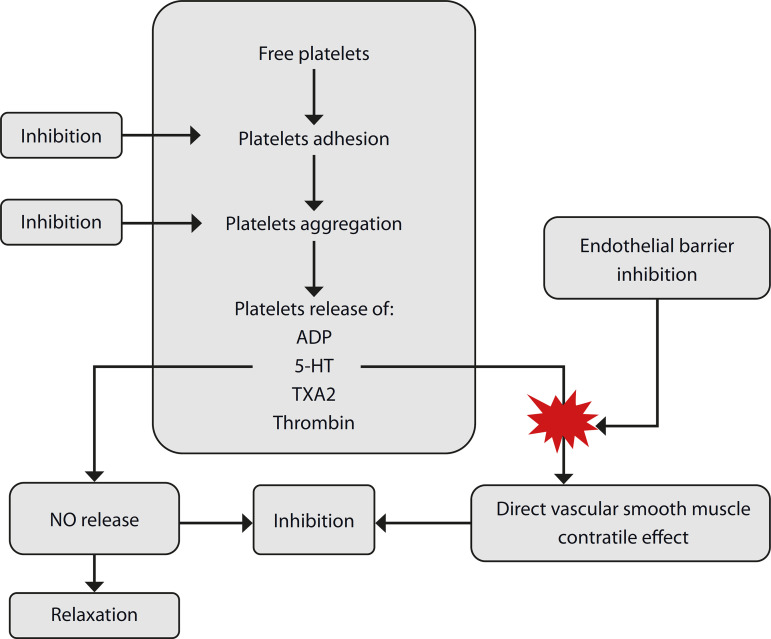
Vasodilator and antiplatelet actions of nitric oxide (NO). 5-HT=5- hydroxytryptamine; ADP=adenosine diphosphate; EDRF=endothelium-derived relaxing factor; TXA2=thromboxane A2

As time went by, the NO saga was associated with spasm and thrombosis due to antiplatelet and vasoconstriction effects (Figure 3). However, in our obsessive struggle against the VS, we always kept in mind that the increased release of NO is also a form of endothelial dysfunction. We proposed a classification to facilitate and allow an overview of endothelial dysfunction (Table 1), but, curiously, we could not start an open discussion on the subject^[4-6]^.

**Table 1:** Classification of endothelial dysfunction.

**Etiological classification**: (A) Primary or *genotypic* endothelial dysfunction: demonstrated in homozygous children with hemocystinuria and normotensive patients with a family history of essential arterial hypertension. (B) Secondary or *phenotypic* endothelial dysfunction: present in all cardiovascular diseases (atherosclerosis, coronary heart disease, high blood pressure, diabetes, and others).
**Functional classification**: (A) *Vasotonic* endothelial dysfunction: present in cardiovascular diseases, which implies a risk of vasospasm and thrombosis. (B) *Vasoplegic* endothelial dysfunction: present in distributive shock states caused by cytokine actions that stimulate the pathological release of relaxing endothelial factors, mainly nitric oxide (sepsis, anaphylaxis, anaphylactoid reactions, and vasoplegia associated with extracorporeal circulation).
**Evolutive or prognostic classification**: (A)* Reversible* endothelial dysfunction: most likely to occur in the early stages of *vasoplegic* dysfunction. (B) *Partly reversible* endothelial dysfunction: to include the idea that it is possible to improve endothelial dysfunction without complete reversal. *Vasotonic* dysfunctions associated with cardiovascular diseases are probably impossible to completely reverse. (C) *Irreversible* endothelial dysfunction: evolution of cardiovascular diseases and sepsis.

MB has been used in clinical practice for many years, namely in the therapy of methemoglobinemia and as a urinary antiseptic, with no contraindications to date regarding its safe use. Thus, its use is already regulated both scientifically and ethically, meaning that there is no encumbrance of lengthy processes of drug regulation and approval. Nowadays, the use of MB seems the most reasonable therapeutic proposal since it does not interfere with NO synthesis and because it is a medication widely used in other clinical conditions. MB’s action is based on the inhibition of guanylate cyclase, preventing the excessive synthesis of cGMP and, consequently, avoiding NO-mediated endothelial relaxation^[1,7]^.

This introduction was intended solely to set the background for the 30 years of the following concepts’ evolution. However, the text’s main target remains as a discussion of vasoplegia as a complication of cardiac surgery.

### The first 15 years - Creating and accumulating concepts

The concepts emerging from the observations obtained during the first 15 years of using MB to treat VS in cardiac surgery have established some critical topics. Nevertheless, we still feel that the cGMP’s importance is underestimated in the literature^[8]^.

**Fig. 4:**
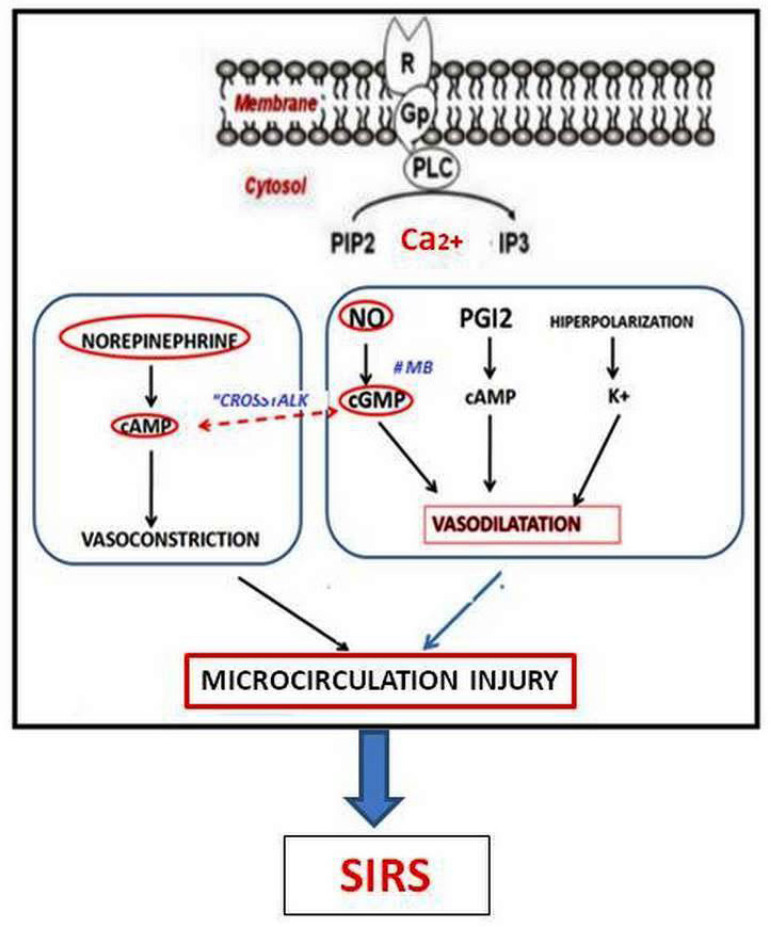
Schematic points of the “crosstalk” between cyclic guanosine monophosphate (cGMP) (nitric oxide [NO]) and 3’,5’-cyclic adenosine monophosphate (cAMP) (prostacyclin). ATP=adenosine triphosphate; Gp=Gp proteins; GS=Gs proteins; GTP=guanosine triphosphate; iNOS=inducible nitric oxide synthase; IP3=inositol triphosphate; MB=methylene blue; PGH2=prostaglandin H2; PGI2=prostacyclin; PIP2=phosphatidylinositol 4,5-biphosphate; PLC=phospholipase C; R=receptor; sGC=soluble guanylyl cyclase

In 2009, we published a personal statement centered at MB as a treatment of VS in cardiac surgery, including 15 years of questions, answers, doubts, and certainties^[1]^. Some observations can be applied to VS: (1) MB is safe at the recommended doses (the lethal dose is 40 mg.kg^-1^); (2) the use of MB does not cause endothelial dysfunction; (3) the MB effect appears in cases of positive NO regulation; (4) MB itself is not a vasoconstrictor because by blocking the cGMP pathway, it releases the 3',5'-cyclic adenosine monophosphate (cAMP) pathway, facilitating the vasoconstrictor effect of epinephrine; (5) MB may act through this mechanism of “crosstalk” (Figure 4) and its use as a first choice medication may not be correct; (6) the most used dosage is 2 mg.kg^-1^ in intravenous (IV) bolus, followed by the same continuous infusion, as plasma concentrations decrease markedly in the first 40 minutes; (7) although there are no definitive multicenter studies, the MB used in the treatment of VS cardiac surgery is currently the best, safest, and cheapest option; (8) however, a possible “window of opportunity” for the effectiveness of MB is not established for humans.

### Twenty years of accumulated experience

Twenty years of accumulated research should now be summarized. In addition to the bibliographical data, some capital questions were elected to consolidate the most important aspects of the MB use in VS treatment associated with cardiac surgery^[1,7]^: (1) What medications would be listed as VS risk factors in cardiac surgery?; (2) Could the systemic inflammatory reaction be caused by blood exposure to the non-endothelial cardiopulmonary bypass circuit?; (3) Is the in vivo MB use safe?; (4) What are the possible complications of MB use?; (5) Does the use of MB cause endothelial dysfunction?; (6) Does the MB injection cause any hemodynamic effect in non-vasoplegic patients?; (7) Does MB have a proper pressure effect?;(8) Does a therapeutic window be established in humans?. We found the responses for most of these questions, but always keeping in mind the real reasons why the cGMP/NO pathway blockage is still underestimated and that most studies on this pathway’s role continue in search of common sense.

### Thirty years of accumulated experience - Keep following the NO saga

**Fig. 5:**
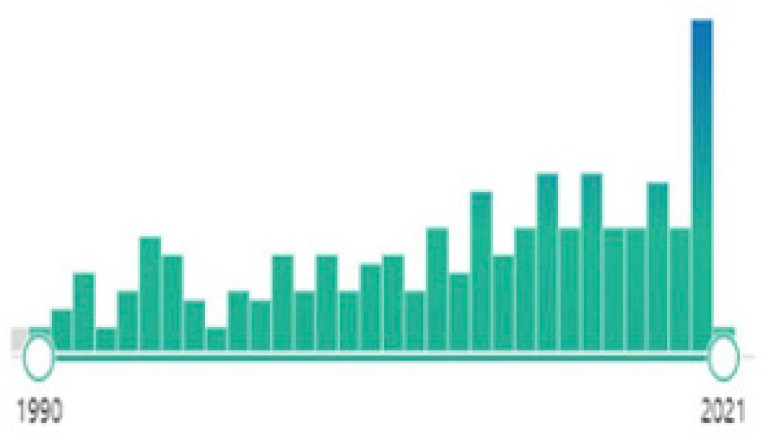
Graph presentation of the increased number of papers as time goes by (Medical Literature Analysis and Retrieval System Online - MEDLINE).

The data in this expanded review leave the impression that the number and quality of publications do not reflect the frequency with which MB is used in clinical practice. Therefore, the difficulty of carrying out multicenter studies is implicit. However, it has been passed on as verbal information. Regarding the increased literature on this topic, one can be optimistic, as shown on the Medical Literature Analysis and Retrieval System Online - MEDLINE database (Figure 5). However, data and medical practices still point to a certainty that the soluble blockade of guanylate cyclase in distributive shock control remains underestimated.

In this environment, the main question arises: “What can we do when circulatory shock becomes refractory to classical therapeutic measures, including administration of fluids, inotropes, and vasoconstrictors?”. The answers to this question are currently limited to the accumulated evidence regarding three cAMP-independent vasoconstriction mechanisms: (1) cGMP/NO-dependent vasoconstriction (the most crucial mechanism); (2) vasopressin administration; and (3) hyperpolarization-dependent vasoconstriction.

One might wonder why these therapeutic alternatives don't always work. We believe that there are at least five aspects to this investigation: (1) lack of consideration of existing guidelines or evidence-based medicine about the accepted treatment options available; (2) the lack of more excellent knowledge of the different vasodilation mechanisms; (3) the possibility of interference between different vasodilation mechanisms; (4) the enzymatic activity of soluble guanylyl cyclase (sGC); and (5) the frequent use of MB as a therapeutic “rescue” or “final” attempt.

Still, many authors are reluctant to recommend MB for early use, given the current minimal level of evidence and possible adverse effects, encouraging studies that systematically collect data to help solve these problems. Similar emergencies involving death risk in cases of circulatory collapse do not allow prospective randomized studies.

Finally, we chose three main points to be highlighted after over 30 years of MB accumulated experience: (1) MB action to treat VS is time-dependent; (2) there is a need for the establishment of the MB therapeutic window in humans; and (3) we believe that MB is a microcirculation protector. This would be the first step towards a systematic guideline to be followed by possible multicenter studies.

Because the present review is considered a unique article to complete a trilogy with the two other articles published in the BJCVS^[1,7]^, which are considerably cited, we will summarize our ongoing main lines of research about the MB role on VS as a relevant complication of the cardiac surgery. We will present a proposal for a therapeutic test with MB, describe the role of MB as a sparer of vasoconstrictor amines with consequent protection of the microcirculation, delineate the use of MB in volume replacement in catastrophic hemorrhages, and discuss the MB use in neonate and children.

### MB test to “keep the pharmacological time” of VS

Considering MB as a “new potential use of an old antidote” to treat distributional shock, one can think that the NO-cGMP pathway plays a central pathophysiological role in the increase in NO and cGMP production, which is well documented in various distributive shock conditions, such as sepsis and anaphylaxis, and there is experimental and clinical evidence to support the use of MB in cases of refractory VS without response to conventional treatment. Perhaps the concept of “window of opportunity” would be best understood by acknowledging that MB's action to treat VS is time-dependent. Despite all of its positive characteristics, when it comes to using MB in VS treatment, the big challenge remains to determine when the inflammation starts and in what “window” is the patient? Thinking about it, we imagine a simple and easy MB system for patients with distributive shock, as follows. Firstly, if there is no hemodynamic monitoring presence, but there is a clear need for an increase in amines to guarantee a minimum mean arterial pressure (MAP) of 65 mmHg, proceed to perform the test with an IV MB bolus infusion (1 mg/kg). The increase in MAP would mean that the patient is in a window dependent on sGC and one should start a regular treatment. If there is hemodynamic monitoring, the results of the test can be quantified. Secondly, in addition to the routine, collect samples for nitrite/nitrate, cGMP, and other inflammatory markers. Thirdly, the use of MB as the last therapeutic option of rescue must be a paradigm to be contested.

If the MB test becomes a practical reality, it is possible that the “window of opportunity” loses its relevance, always keeping in mind that determination of the starting point of inflammatory reaction is a challenging task.

### Combination of broad spectrum vasopressors, vasopressor-sparing strategies, and microcirculation protection

We continue to lose the fight against vasodilatory shock due to the exacerbated inflammatory response. Volume restoration and vasopressors are not always able to overcome this situation, requiring new therapeutic strategies. In a recent Letter to the Editor^[9]^, we suggested that the possibility of a combination of three concepts would be an exciting idea. These three concepts are (1) the broad spectrum vasopressors, (2) the vasopressor-sparing strategies, and (3) microcirculation protection.

The concept of broad spectrum vasopressors suggests that the septic shock treatment must be started on multiple vasopressors with a different mechanism of action. Catecholamine vasopressor support-sparing strategies is a mandatory concept to pursue adjunctive therapeutic options to reduce vasoactive support requirements without compromising arterial pressure and the microcirculation^[10]^ and reduce the need for high doses of catecholamine vasopressors. It would also be desirable to seek new vasopressors that increase the arterial blood pressure without microcirculatory damage.

### Possible MB role as microcirculation protector

**Fig. 6:**
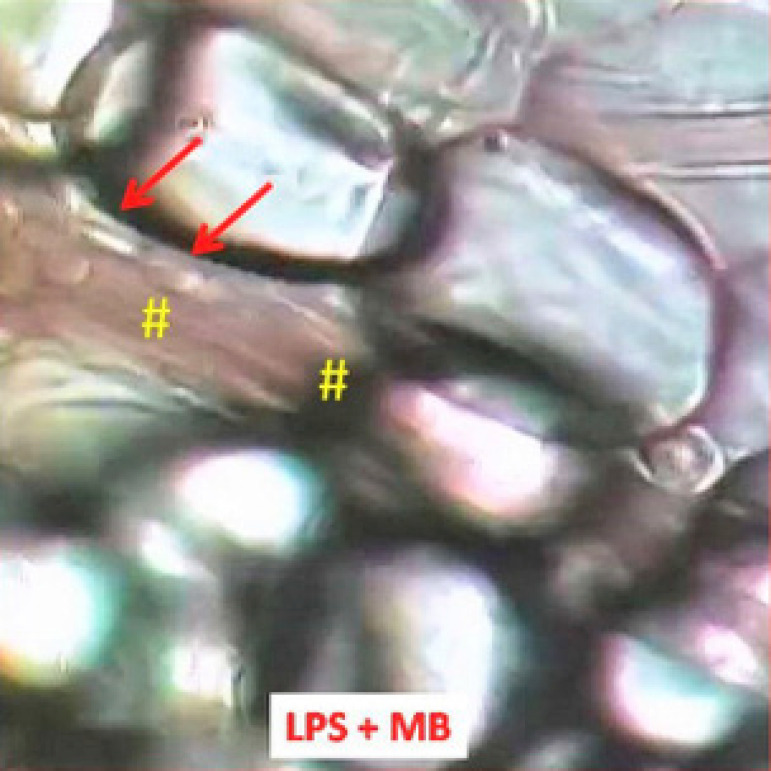
Photograph of rat mesenteric microcirculation showing the excellent structure after Escherichia coli lipopolysaccharide injection. The # shows the venula; the arrows show almost normal leukocyte adhesion. LPS=; MB=methylene blue

MB has been used with success as a treatment for vasopressor-refractory septic shock vasoplegia. The supposed MB mechanism is the inhibition of the microvasculature of the endothelial NO and improved responsiveness to amines. However, to date, only one relevant study has demonstrated the microcirculatory MB effect^[11]^. Also, few data show the impact on global hemodynamics and microcirculation in an experimentally induced rat septic shock model (Figure 6). We investigated with the aid of intravital microscopy, confirming Nantais data^[11]^.

The therapeutic use of NOS including humans has been abandoned based on increased mortality due to microcirculatory damage. So, using intravital microscopy, we decided to test an exciting hypothesis that MB should protect the microcirculation and bring back the NOS inhibition to the clinical scenario. Unfortunately, we found that MB does not protect the microcirculation against the deleterious effects of the NOS inhibitor (results not published).

Also, there are several limitations of using microcirculation parameters in clinical practice and shock resuscitation. However, several measurements are suggested to represent microcirculation, including lactate levels, the gap between the venous and arterial carbon dioxide, and capillary filling time.

### MB in severe bleeding and MB use in child and neonates

These two aspects are being studied in our laboratory, and our data are compatible with the safe MB use. The early treatment of traumatic hemorrhagic shock is mandatory, and it is a research challenge to determine the best way to prevent cardiac arrest before bleeding control. The blockade of the cGMP/NO pathway, although its pathophysiological justification, has not been considered in clinical or experimental practice. Our laboratory data showed the MB safety to be used earlier on significant hemorrhages.

**Table:** 

Authors' roles & responsibilities	
PRBE	Substantial contributions to the conception or design of the work; or the acquisition, analysis, or interpretation of data for the work; drafting the work or revising it critically for important intellectual content; agreement to be accountable for all aspects of the work in ensuring that questions related to the accuracy or integrity of any part of the work are appropriately investigated and resolved; final approval of the version to be published
ROSS	Substantial contributions to the conception of the work; drafting the work or revising it critically for important intellectual content
SB	Drafting the work or revising it critically for important intellectual content; final approval of the version to be published
MAM	Substantial contributions to the conception of the work; agreement to be accountable for all aspects of the work in ensuring that questions related to the accuracy or integrity of any part of the work are appropriately investigated and resolved
FLSS	Drafting the work or revising it critically for important intellectual content; final approval of the version to be published
ABF	Drafting the work or revising it critically for important intellectual content; final approval of the version to be published
