# ERRATUM

**DOI:** 10.21470/1678-9741-2021-0955e

**Published:** 2022

In the article “After Thirty Years, We Still Cannot Understand Why Methylene Blue is not a Reference to Treat Vasoplegic Syndrome in Cardiac Surgery”, with DOI code http://dx.doi.org/10.21470/1678-9741-2021-0955 published in the Brazilian Journal of Cardiovascular Surgery, 36.3, the surnames of the fourth, fifth and sixth authors are misspelled:

In the original version the information was:

Paulo Roberto B. Evora^1^, MD, PhD; Ricardo O. S. Soares^2^, MD; Solange Bassetto^1^, MD; Maria Auxiliadora Martins^1^, MD, PhD; Fábio Luis da Silva Silva^1^, MD; Anibal Basile Filho^1^, MD, PhD

The correct information is:

Paulo Roberto B. Evora^1^, MD, PhD; Ricardo O. S. Soares^2^, MD; Solange Bassetto^1^, MD; Maria Auxiliadora-Martins^1^, MD, PhD; Fábio Luis-Silva^1^, MD; Anibal Basile-Filho^1^, MD, PhD

